# Healing of the epithelial barrier in the ileum is superior to endoscopic and histologic remission for predicting major adverse outcomes in ulcerative colitis

**DOI:** 10.3389/fmed.2023.1221449

**Published:** 2023-10-10

**Authors:** Timo Rath, Raja Atreya, Julia Bodenschatz, Wolfgang Uter, Carol I. Geppert, Francesco Vitali, Sebastian Zundler, Maximilian J. Waldner, Arndt Hartmann, Markus F. Neurath

**Affiliations:** ^1^Department of Gastroenterology, Ludwig Demling Endoscopy Center of Excellence, University Hospital Erlangen, Friedrich-Alexander University Erlangen-Nuernberg, Erlangen, Germany; ^2^Institute for Medical Informatics, Biometry and Epidemiology, Friedrich-Alexander University Erlangen-Nuernberg, Erlangen, Germany; ^3^Institute for Pathology, University Hospital Erlangen, Friedrich-Alexander University Erlangen-Nuernberg, Erlangen, Germany; ^4^Deutsches Zentrum für Immuntherapie DZI, Friedrich-Alexander University Erlangen-Nuernberg, Erlangen, Germany

**Keywords:** inflammatory bowel diseases, endoscopy, histology, intestinal barrier, ulcerative colitis

## Abstract

**Background:**

Achieving endoscopic remission is a key therapeutic goal in patients with ulcerative colitis (UC) that is associated with favorable long-term disease outcomes. Here, we prospectively compared the predictive value of endoscopic and/or histologic remission against ileal barrier healing for predicting long-term disease behavior in a large cohort of UC patients in clinical remission.

**Methods:**

At baseline, UC patients in clinical remission underwent ileocolonoscopy with assessment of ileal barrier function by confocal endomicroscopy. Endoscopic and histologic disease activity and ileal barrier healing were scored using validated scores. During subsequent follow-up (FU), patients were closely monitored for clinical disease activity and occurrence of major adverse outcomes (MAO) defined as the following: disease relapse; UC-related hospitalization; UC-related surgery; necessity for initiation or dose escalation of systemic steroids, immunosuppressants, small molecules or biological therapy.

**Results:**

Of the 73 UC patients included, 67% experienced MAO during a mean FU of 25 months. The probability of MAO-free survival was significantly higher in UC patients with endoscopic and/or histologic remission compared to patients with endoscopically and/or histologically active disease. Ileal barrier healing on endomicroscopy was highly accurate for predicting the further course of UC and outcompeted endoscopic and histologic remission for predicting MAO-free survival.

**Conclusion:**

Ileal barrier healing in clinically remittent UC patients can accurately predict future MAO development and is superior in its predictive capabilities than endoscopic and histologic remission. Ileal barrier healing therefore represents a novel and superior surrogate parameter for stratification of UC patients according to their risk for development of complicated disease behavior.

**Clinical trial registration:**

https://classic.clinicaltrials.gov/ct2/show/NCT05157750, identifier NCT05157750.

## Introduction

Ulcerative colitis (UC) patients that achieve endoscopic remission have a more favorable course of disease with decreased flaring of disease and increased rates of steroid free clinical remission and colectomy-free survival as compared to UC patients without endoscopic remission. Therefore, achieving endoscopic remission in patients with ulcerative colitis is a major treatment goal that is advocated by several guidelines for clinical practice and for trial endpoints (1–5). In addition to endoscopic remission, histologic remission is another emerging endpoint in patients with ulcerative colitis that is associated, as consolidated by several meta-analyses (6–8), with better disease outcome compared to clinical remission and/or endoscopic healing. However, assessing histological remission UC is complex with currently 26 different histopathological scores out of which only two are validated (9). Furthermore, although acknowledged as a sensitive measure of inflammation, the STRIDE working group does not recommend histologic remssion as a formal treatment target in UC (4).

Just recently, we compared the value of endoscopic remission and histologic remission against the integrity of the intestinal barrier for predicting long-term disease behavior in clinically remittent IBD patients for predicting major adverse outcomes (MAO). In this ERIca trial (Erlangen Remission in IBD), a large cohort of IBD patients in clinical remission were prospectively included and closely monitored during long term follow for more than 2 years and this study provided first evidence that assessing the integrity of the intestinal barrier with confocal laser endomicroscopy (CLE) can not only accurately predict disease behavior but also that intestinal barrier healing is superior compared to endoscopic and histologic remission for predicting MAOs (10). However, in the ERIca trial, we only analyzed colonic barrier function for predicting disease behavior in UC. Therefore, we now aimed to extend these observations and to explore whether assessment of ileal barrier function in patients with ulcerative colitis can predict the occurrence of major adverse outcomes in clinically remitted patients with ulcerative colitis.

### Study design and participants

This study was an extended analysis of data from the ERIca trial which was conducted at the Ludwig Demling Endoscopy Center of Excellence and the IBD outpatient department at the University Hospital of Erlangen as a prospective observational study (10). The study was approved by the local ethics committee as well as the Institutional Review Board of the Medical Faculty of the Friedrich-Alexander University Erlangen-Nuremberg. After written informed consent was obtained, patients with an established diagnosis of UC for at least 12 months and which presented in clinical remission were enrolled. Exclusion criteria were as follows: poor bowel preparation, total colectomy, concomitant beta blocker therapy, known allergy to fluorescein or a planned change in IBD-related pharmacotherapy. Clinical disease activity was assessed along the Mayo clinical disease activity score (MCS) prior to study inclusion (11). After ileocolonoscopy with confocal laser endomicroscopy, close meshed followed up in our IBD outpatient department every 4 to 8 weeks for patients under biological therapy and every 8 weeks for patients under conventional therapy was performed. At each visit, clinical disease activity using the MCS along with routine laboratory parameters and current and past medications were assessed. Furthermore, major adverse outcomes (MAO), defined as the following, were recorded at each visit: (i) disease relapse; (ii) UC-related hospitalization, (iii) UC-related surgery, (iv) necessity for initiation or dose escalation of systemic steroids, immunosuppressants, small molecules or biological therapy.

### Colonoscopy and confocal laser endomicroscopy

Bowel preparation was performed with low-volume PEG-based bowel lavage in a split dose regimen in all patients scheduled for ileocolonoscopy. In case the patients were scheduled for sigmoidoscopy only, the patients received dihydrogen dihydrate enema prior to sigmoidoscopy. According to consensus statements, endoscopic remission and/or healing during WLE were defined in the following way (3, 12): Endoscopic remission, Mayo Endoscopy Score (MES) ≤1; Endoscopic healing, MES = 0 (13, 14). Representative endoscopic images of patients with and without endoscopic remission are shown in Supplementary Figure S1.

Confocal Laser Endomicroscopy was performed as previously described (10). After reaching the terminal ileum, 5 mL Fluorescein 10% were intravenously injected as a contrast agent. Afterwards, the CLE probe was positioned under endoscopic guidance onto the mucosa of the terminal ileum, low-powered blue laser light of a wavelength of 488 nm was activated for tissue illumination by the hit of a foot pedal and a CLE video of approximately 2 min was recorded with an image acquisition rate of 8 frames per second. All CLE images for each patient were stored on an external hard drive and were independently reviewed for presence of ileal barrier dysfunction by three expert readers (T.R., J.B., F.V.) blinded to the clinical results of the patients.

Barrier dysfunction in the terminal ileum was assessed using the semi-quantitative Watson score into three grades as previously described (10, 15–20): (I) *intact epithelial barrier* with no fluorescein leakage, (II) *functional barrier defect* with shedding of single epithelial cells and fluorescein leakage into the intestinal lumen, (III) *structural barrier defect* with shedding of multiple epithelial cells, exposure of the lamina propria to the lumen and fluorescein leakage into the lumen. The different grades of ileal barrier (dys)function as assessed by CLE are shown in Supplementary Figure S2.

### Histologic analysis

From each patient, samples for histopathology were obtained at the sites where CLE imaging was performed. In addition, in case macroscopic inflammation was present during WLE, these areas were also biopsied matching those areas that were also examined by CLE. All samples were scored by an experienced GI pathologist (A.H.) blinded to clinical and endoscopic patient data. For histopathological scoring in UC, Robarts Histopathology Index (RHI) [24] as well as Nancy histological index (NHI) [25] were used as validated histology scores. Histologic disease remission was defined as a RHI ≤ 3 without lamina propria or epithelial neutrophils or a NHI ≤ 1. Representative histolopathologic images are shown in Supplementary Figure S3.

### Endpoints, sample size and statistical analysis

The primary endpoint of this study was to compare the predictive values of ileal barrier healing, endoscopic remission and histologic remission for predicting occurrence of MAO in UC patients. Statistical analyzes were performed using the R statistical software package, version 4.0.x.^1^ All statistical tests were considered explorative without alpha adjustment. Moreover, Kaplan–Meier analysis was performed to examine the time to the occurrence of MAOs (or censoring at end of follow-up).

## Results

### Study inclusion and clinical patient characteristics

Between 2017 and 2019, a total of 81 UC patients were included in the study. From these 81 patients, 73 patients had valid and complete information regarding the occurrence of MAOs during follow-up and data on endoscopic remission and healing, histologic remission and barrier function in the terminal ileum were available for all patients. Clinical, endoscopic and histological characteristics of the UC patient cohort are summarized in Table 1.

**Table 1:** Clinical, endoscopic and histologic characteristics of the UC patient cohort.

Ulcerative colitis (*n* = 73)	
*Clinical characteristics*	
Age (y)	
Mean, range	38.3 (18–69)
Sex (m/f)	36/37
BMI	
Mean, range	25.6 (17.2–39.2)
Disease duration (y)	
Mean ± SD	9 ± 7.6
Extent of disease, *n* (%)	
Proctitis	5 (6.8)
Leftsided colitis	34 (46.6)
Pancolitis	34 (46.6)
Extraintestinal manifestations, *n* (%)	18 (24.7)
Primary sclerosing cholangitis, *n* (%)	2 (2.7)
*Medication, n (%)*	
5-ASA derivates	
Mesalazin	12 (16.4)
Corticosteroids	
Budesonide (with colonic delivery)	2 (2.7)
Prednisolone (n)	2 (2.7)
Mean dose (mg) ± SD	10 ± 4
Immunomodulator	
6-Mercaptopurin	1 (1.4)
Azathioprin	3 (4.1)
Biological therapy	
Anti-TNF	28 (38.4)
Vedolizumab	11 (15.1)
Tofacitinib	3 (4.1)
Ustekinumab	2 (2.7)
Combination therapy	5 (6.8)
No medication	4 (5.5)
*Laboratory parameters, mean ± SD*	
Leukocyte count (10^9^/L)	7.9 ± 3.3
C-reactive Protein (mg/L)	5.0 ± 8.3
Hematocrit (%)	41.5 ± 4.1
*Endoscopic and histopathologic data*	
Mayo endoscopic score, *n* (%)	
≤ 1	41 (56.2)
> 1	32 (43.8)
Barrier function, *n* (%)	
Ileum	
Barrier healing present	22 (30.1)
Histopathology scoring, *n* (%)	
RHI ≤ 3	41 (56.2)
RHI > 3	32 (43.8)
Nancy <1	39 (53.4)
Nancy ≥1	34 (46.6)
Follow up (FU)	
Mean ± SD (months)	26 ± 12
Patients without MAO during FU, *n* (%)	24 (32.9)

RHI, Robarts Histology Index; Nancy, Nancy Histological Index; MAO, major adverse outcomes: disease flare; UC-related hospitalization; UC-related surgery; necessity for initiation or escalation of systemic steroids, immunosuppressants, small molecules or biological therapy.

From these 73 UC patients included in the final analysis, 41 (56.2%) patients had endoscopic remission on WLE, as defined by a MES ≤ 1, at study inclusion (Table 1). Histologic remission, as defined by RHI and NHI, was observed in 56.2 and 53.4% patients, respectively, during baseline endoscopy. In 34 UC patients (44.4%), the combination between endoscopic and histologic remission (as assessed by the RHI), was present. In contrast, barrier healing in the ileum was observed in only 22 UC patients in the terminal ileum (30.1%) during baseline endoscopy. Detailed clinical, endoscopic and histologic characteristics in UC patients with and without ileal barrier healing are comparatively displayed in Supplementary Table S1. In additional studies, we determined levels of serum zonulin, as a marker that has been used in a variety of studies to assess integrity of the intestinal barrier, and noted that serum zonulin levels did not significantly differ between UC patients with intact ileal barrier as compared to those with ileal barrier dysfunction (Supplementary Figure S4).

### Follow up and occurrence of major adverse outcomes in UC patients

Mean follow up in UC patients was 26 months (Table 1). In 24 UC patients, no MAOs occurred in the course of follow up, while in the remaining 49 patients it was, with a mean time lag for MAO occurrence of 3.2 months (SD ± 2.5 months, range 1–10 months) from baseline endoscopy.

**Figure 1:**
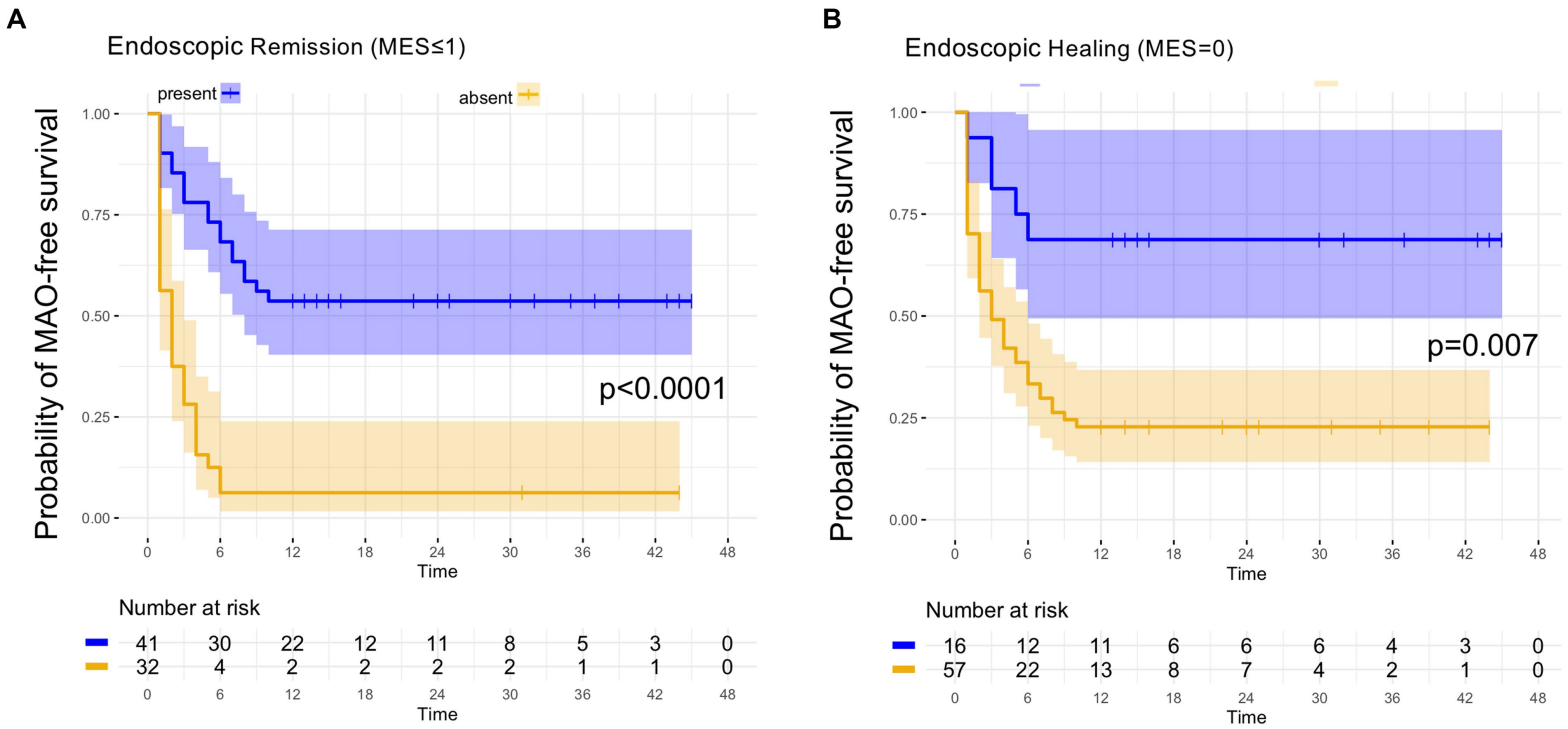
Kaplan–Meier analyzes for the occurrence of major adverse outcomes in UC patients with endoscopic remission and endoscopic healing. **(A)** In UC patients with endoscopic remission, as defined by an MES ≤ 1, the probability of remaining free of major adverse outcomes (MAO) during FU was significantly higher compared to patients with endoscopically active disease (MES > 1). **(B)** UC patients with endoscopic healing, as defined by an MES = 0, exhibited a significantly higher likelihood of remaining without MAO during FU as compared to UC patients with a MES > 0.

The MAO rates in patients with endoscopic and histologic remission and in patients with barrier healing are summarized in Table 2. As shown in Table 2, of the 41 patients with endoscopic remission at study inclusion, 19 developed MAOs during FU, leading to a MAO rate for endoscopic remission of 46.3%. Time to event analysis using Kaplan–Meier estimates showed that UC patients with endoscopic remission had a significantly higher probability of remaining free of MAOs during FU compared to those patients with endoscopically active disease (*p* < 0.0001, Figure 1A). When applying a more stringent endoscopic definition considering only patients with a MES = 0 (i.e., endoscopic healing), a total of 16 UC patients exhibited endoscopic healing. Of these, 5 experienced MAO during the course of follow-up, leading to a MAO rate in patients with MES = 0 of 31.3% (Table 2). Correspondingly, the probability for MAO-free survival during FU was significantly higher in UC patients with endoscopic healing as compared to those with a MES > 0 (*p* = 0.007, Figure 1B).

**Table 2:** Major adverse outcome (MAO) rates in patients with ulcerative colitis.

Parameter	MAO rate
Endoscopic remission	46.3% (19/41)
Endoscopic healing	31.3% (5/16)
RHI histologic remission	48.8% (20/41)
NHI histologic remission	46.2% (18/39)
Endoscopic remission + RHI histologic remission	41.2% (14/34)
Barrier healing ileum	9.1% (2/22)

MAO, major clinical events (disease flare; IBD-related hospitalization; IBD-related surgery; necessity for initiation or escalation of systemic steroids, immunosuppressants, small molecules or biological therapy) Endoscopic remission, MES ≤ 1; Endoscopic healing, MES = 0; RHI Histologic remission, RHI ≤ 3 without lamina propria or epithelial neutrophils; NHI Histologic remission, NHI ≤ 1.

**Figure 2:**
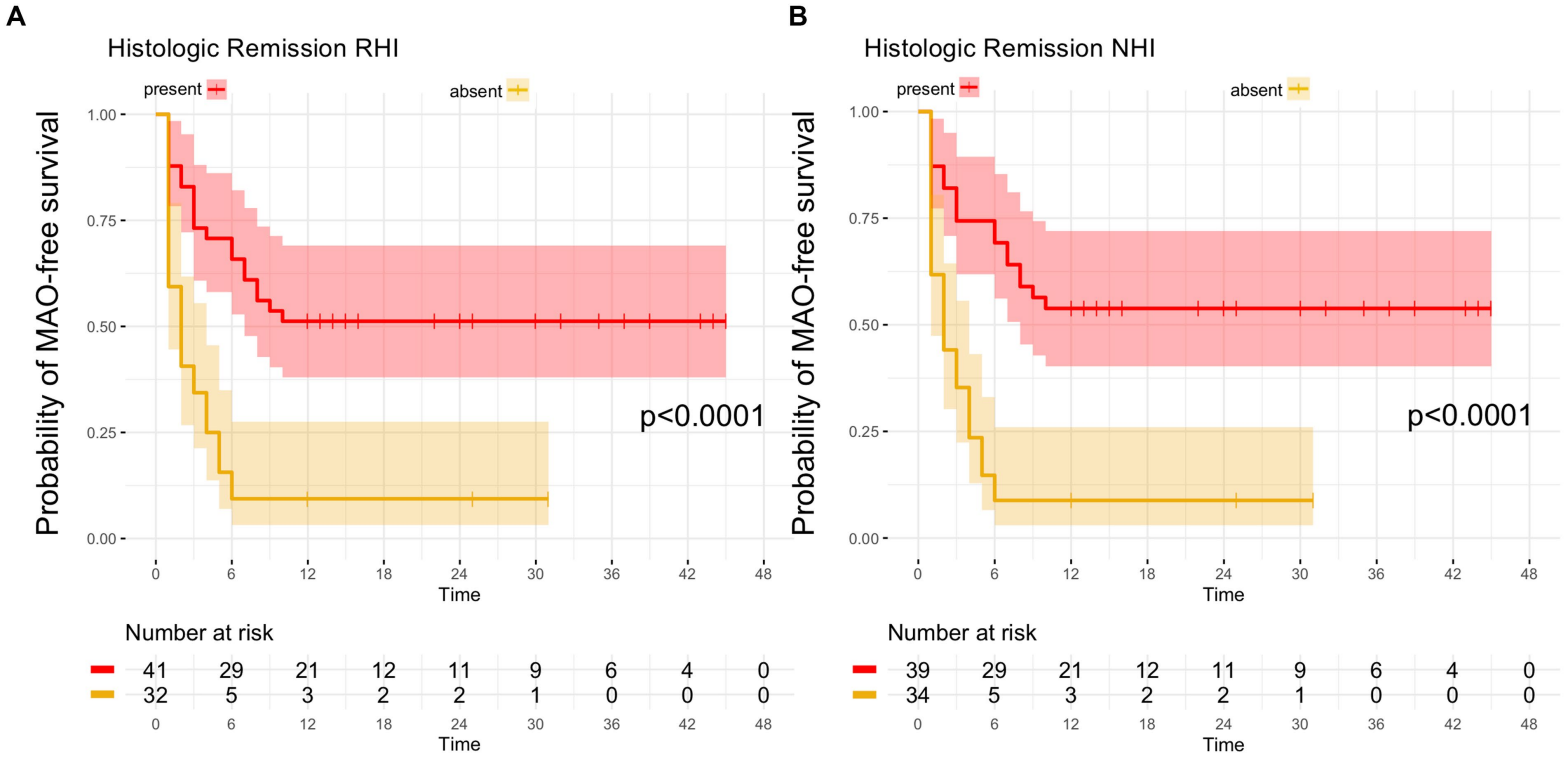
Kaplan–Meier analyzes for the occurrence of major adverse outcomes in UC patients with histologic remission. **(A)** In UC patients with histologic remission, defined by a Robarts Histology Index (RHI) ≤ 3, the probability of remaining free of major adverse outcomes (MAO) during FU was significantly higher compared to patients with histologically active disease (RHI > 3). **(B)** UC patients with histologic remission, as determined by a Nancy Histology Index (NHI) ≤1, exhibited a significantly higher likelihood of remaining without MAO during FU as compared to UC patients with an NHI > 1.

**Figure 3:**
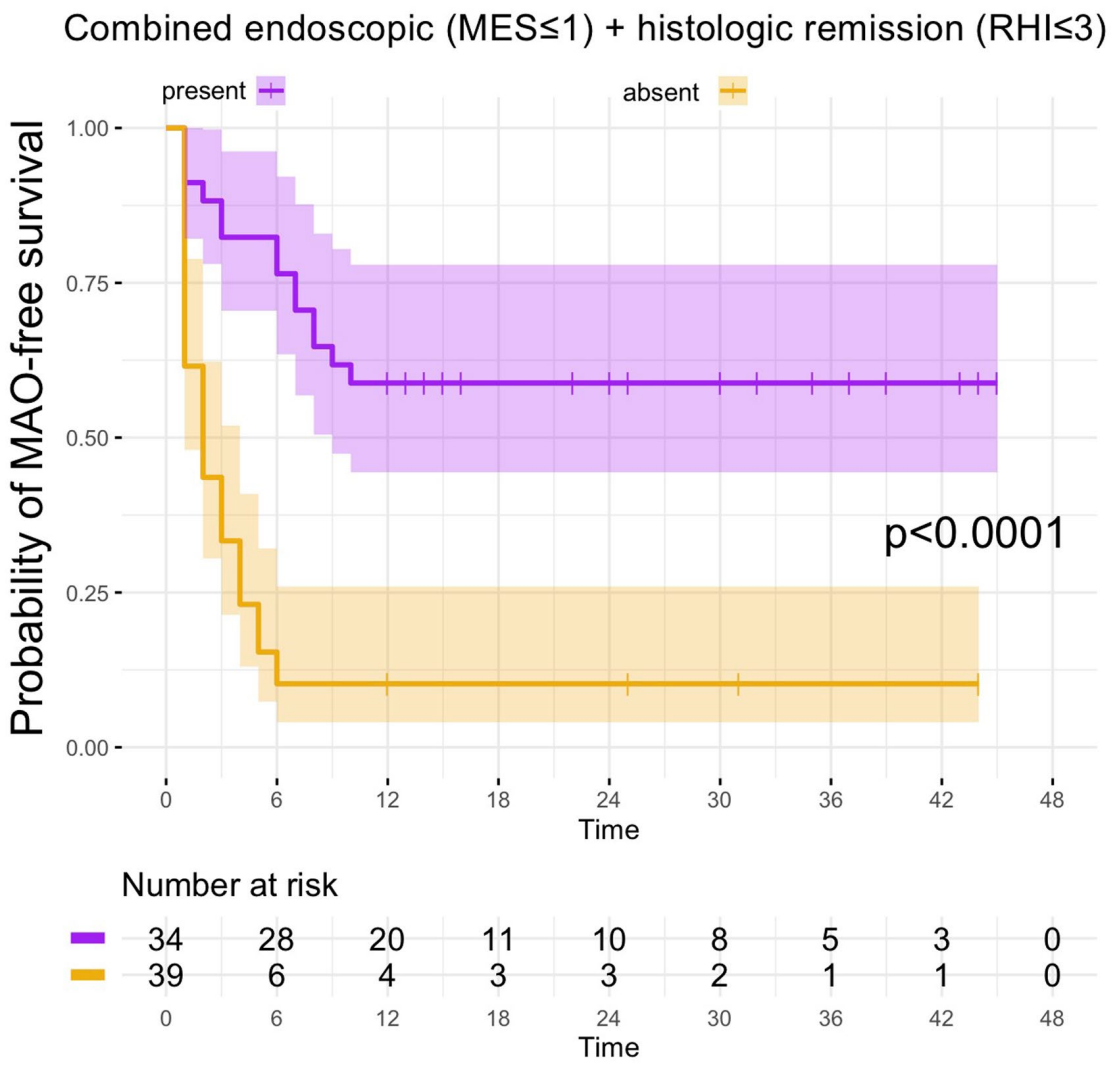
Kaplan–Meier analyzes for the occurrence of major adverse outcomes in UC patients with combined endoscopic and histologic remission. In UC patients, in which the combination between endoscopic and histologic remission was present, the probability of MAO-free survival was significantly higher compared to UC patients without combined endoscopic and histologic remission.

From the 41 UC patients with histologic remission as defined by the RHI, 20 developed MAO during follow-up (RHI MAO-rate: 48.8%) while in 18 out of 39 patients with histologic remission as defined by the NHI, MAO occurred during the course of follow-up (NHI MAO-rate: 46.2%). On Kaplan–Meier analysis, patients with histologic remission along the RHI and the NHI were significantly more likely to remain MAO-free during FU as compared to UC patients with histologically active disease (both *p* < 0.0001, Figure 2). From those 34 patients with combined histologic (as defined by the RHI) and endoscopic remission, 14 experienced MAOs during study follow-up (MAO rate: 41.2%) and likewise, those patients with combined endoscopic and histologic remission had a significantly better course of disease in terms of remaining free of MAO on Kaplan–Meier estimates (*p* < 0.0001, Figure 3).

**Figure 4:**
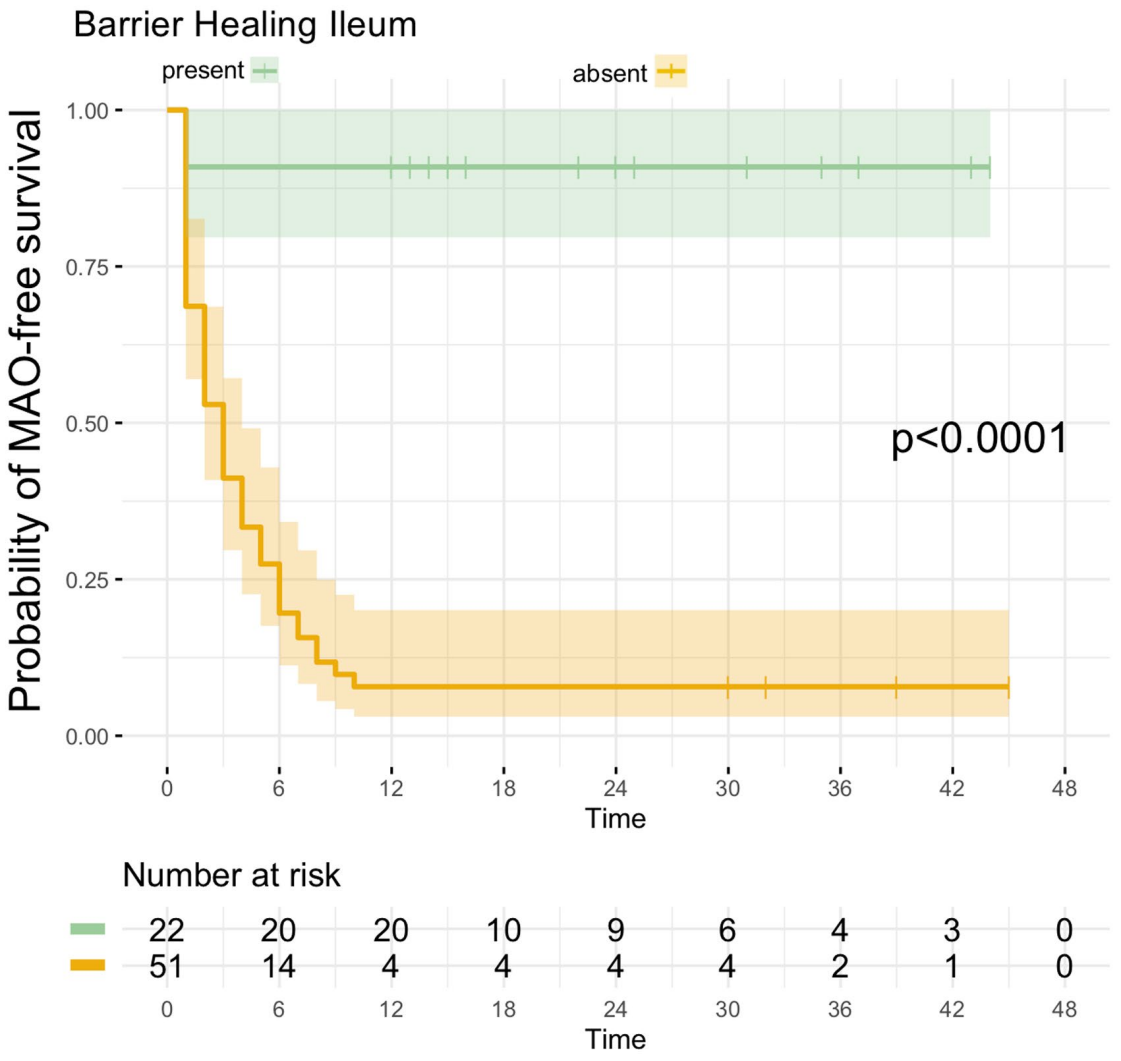
Kaplan–Meier analyzes for the occurrence of major adverse outcomes in UC patients with ileal barrier healing. UC patients with ileal barrier healing exhibited a significantly higher likelihood of remaining free of MAO during FU as compared to UC patients in which ileal barrier dysfunction was present during baseline endoscopy.

Of the 22 UC patients with barrier healing in the terminal ileum, only 2 patients developed MAO during FU, hence MAO rate in patients with ileal barrier healing was 9.1% (Table 2). Consistent with this, UC patients with barrier healing in the terminal ileum had a significantly more favorable course of disease as shown by Kaplan–Meier analysis (p < 0.0001, Figure 4).

### Diagnostic performances of endoscopic healing, histologic healing and barrier healing for the prediction of the course of disease

Based on the low MAO rates in UC patients with intact ileal barrier and the high probabilities for remaining without MAOs during follow up, we further set off to directly compare the diagnostic performances of endoscopic and histologic remission as established parameters against ileal barrier healing for the prediction of long-term disease outcome.

Endoscopic remission (MES ≤ 1), had an overall accuracy of 71.2% for predicting the further course of disease with positive and negative predictive values of 53.7 and 93.8%, respectively (Table 3). Considering only patients with endoscopic healing (MES = 0), the accuracy for predicting MAO-free course of disease was increased with an accuracy of 75.3% and positive and negative predictive values of 68.8 and 77.1%, respectively (Table 3).

**Table 3:** Diagnostic performances of endoscopic remission, histologic remission and ileal barrier healing for predicting major adverse outcomes in UC patients.

Parameter	Accuracy (95% CI-Interval)	Sensitivity (95% CI-Interval)	Specificity (95% CI-Interval)	PPV (95% CI-Interval)	NPV (95% CI-Interval)
Endoscopic remission (MES ≤ 1)	71.2% (59.5–81.2%)	91.7% (73–99%)	61.2% (46.2–74.8%)	53.7% (44.4–62.7%)	93.8% (79.6–98.3%)
Endoscopic healing (MES = 0)	75.3% (63.9–84.7%)	45.8% (25.6–67.2%)	89.8% (77.8–96.6%)	68.8% (46.3–84.9%)	77.1% (69.8–83.2%)
Robarts histologic remission^a^	68.5% (56.6–78.9%)	87.5% (67.6–97.3%)	59.2% (44.2–73%)	51.2% (42.1–60.3%)	90.6% (76.6–96.6%)
Nancy histologic remission^b^	71.2% (59.5–81.2%)	87.5% (67.6–97.3%)	63.3% (48.3–76.6%)	53.9% (44–63.5%)	91.2% (77.8–96.8%)
Endoscopic remission (MES ≤ 1) + Robarts histologic remission^a^	75.3% (63.9–84.7%)	83.3% (62.6–95.3%)	71.4% (56.7–83.4%)	58.8% (47–69.7%)	89.7% (77.9–95.6%)
Barrier healing ileum	91.8% (83–96.2%)	83.3% (62.6–95.3%)	95.9% (86–99.5%)	90.9% (71.8–97.5%)	92.2% (82.7–96.6%)

PPV, positive predictive value; NPV, negative predictive value; CI, confidence interval.

a Histologic remission according to the Robarts’ histology index.

b Histologic remission according to the Nancy histology index.

Histologic remission, as defined by the RHI and the NHI, exhibited an accuracy of 68.5 and 71.2%, respectively, with comparable positive and negative predictive values of the two histopathology scores for predicting the occurrence of MAO (Table 3).

Using the combination of endoscopic remission (as defined by a MES ≤ 1) and histologic remission as assessed by the RHI, overall accuracy for predicting the occurrence of major clinical events was increased to 75.3% with a positive and negative prediction of 58.8 and 89.7%, respectively (Table 3).

In contrast, the diagnostic performance of ileal barrier integrity as a new surrogate parameter for the prediction of long-term disease behavior was increased compared to the aforementioned parameters. In this regard, barrier healing in the terminal ileum had an overall accuracy of 91.8% with a positive and negative prediction of 90.9 and 92.2% (Table 3) and was therefore clearly superior in its predictive capabilities compared to the other parameters.

## Discussion

Increased intestinal permeability in IBD patients was first noted already more than 30 years ago and found to predict clinical relapse in CD patients in remission (21, 22) and even earlier evidence on relatives of CD patients already suggested that increased intestinal permeability is not secondary to clinically manifest intestinal inflammation but rather constitutes a primary defect that is etiologically involved in disease pathogenesis (23).

In addition to this almost historic evidence, a just recently published study assessed intestinal permeability by the lactulose-mannitol-ratio (LMR) in over 1,400 asymptomatic first-degree relatives of CD patients. Importantly, as observed during long term follow up, increased LMR as a marker of increased intestinal permeability acted as an independent risk factor for developing Crohn’s disease in first degree relatives in the future conferring a 3-fold risk increase (24).

Several studies have already used CLE for dynamic visualization and assessment of intestinal barrier integrity. Published already a decade ago, Kiesslich and co-workers were able to show that in CD and UC patients in clinical remission increased cell shedding with fluorescein leakage in the ileum, as visualized with CLE, is associated with subsequent disease relapse within 12 months after endomicroscopic examination. Importantly, in this study a novel scoring system for semiquantitative grading system (the “Watson-Score”) of ileal barrier dysfunction was devised that exhibited a specificity >90% for predicting subsequent disease flare in clinically remittent IBD patients. Using this score, these results were subsequently corroborated in an independent cohort of IBD patients by Karstensen and co-workers. In this study, a Watson-Score of 2 or 3, representative of functional or structural ileal barrier dysfunction, exhibited a sensitivity of 89% for predicting disease relapse within the next 12 months in clinically remittent CD patients (16). Another prospective study on 110 IBD patients with endoscopic mucosal healing was able to establish an association between impaired intestinal permeability, as assessed by quantitative grading of barrier dysfunction by CLE, and persistence of clinical symptoms. Importantly, increases in intestinal permeability in the ileum directly correlated with severity of diarrhea in both, UC and CD patients and led the authors to speculate that resolution of mucosal permeability beyond mucosal healing might improve outcomes of patients with IDB (15).

Just recently, we reported the results of our ERIca trial in which we compared the value of endoscopic and histologic remission against intestinal barrier healing for predicting the further course of disease in a large cohort of clinically remitted IBD patients (10). As shown in this trial, in CD patients barrier healing in the ileum and colon by far outcompeted endoscopic and histologic remission in forecasting the further course of disease during close-meshed multiannual follow-up. In UC patients, we observed that barrier healing in the colon was also associated with decreased risk of development of major adverse outcomes with superior predictive performance compared with endoscopic and histologic remission. However, in the ERIca trial, we did not analyze barrier function in the ileum for predicting disease behavior in UC patients.

Therefore, against the background of published reports on the relevance of ileal barrier function in UC (15, 17), we now aimed to extend the observations of the ERIca trial and explored whether assessment of ileal barrier function in patients with UC can likewise be used to predict the occurrence of major adverse outcomes in clinically remitted UC patients. Our results show that ileal barrier healing is indeed related with favorable disease outcome: of the 73 UC patients included, 22 patients exhibited ileal barrier healing and of these 22 patients with barrier healing, only 2 patients developed major adverse outcomes during a mean follow-up period of 26 months. Consistent with this, time-to-event analysis using Kaplan Meier estimates showed that ileal barrier healing was associated with a significantly more favorable course of disease over a mean follow-up period of 26 months in clinically remittent UC patients. In addition to that, our data clearly indicate that ileal barrier is superior to endoscopic or histologic remission, or the combination of the later. As such, the MAO rate was by far lower for ileal barrier healing as compared to endoscopic or histologic remission and the diagnostic accuracy of ileal barrier healing for forecasting the further course of disease outcompeted those of endoscopic or histologic remission or their combination.

In parallel to these clinical data strengthening the relevance of impaired function in IBD patients, basic science has identified impairments in tight junctions and epithelial resistance in both, UC and CD (25–27). In their togetherness, impaired barrier function leading to increased intestinal permeability is increasingly recognized a key etiologic factors in the development of IBD (28). Based on our observation that ileal barrier healing is highly predictive for more favorable disease outcome in UC, which has been commonly defined as a disease confined to the colon and the rectum, the following aspects are worth considering: (i) although traditionally regarded as two distinct diseases with clear distinction between UC and CD, emerging evidence suggests that IBD is more and more perceived as a continuous spectrum. As such, already a decade ago whole genome gene expression meta-analysis in IBD demonstrated a lack of major differences between Crohn’s disease and ulcerative colitis (29) and aggregated genetic risk scores representing the cumulative burden of mutations in known IBD risk loci introduced the concept of a disease spectrum along the disease location axis (30) and (ii) the affection of the terminal ileum in patients with UC is increasingly recognized as a further disease manifestation that is different from clinical evident backwash ileitis. As such the existence of ulcerations in the terminal ileum without co-existing evidence of backwash ileitis in UC patients have been described with varying frequencies (31) and a recent review proposed that ileal inflammation in UC represents a primary manifestation of UC which has been referred to as “UC-associated ileitis” (iii) we and others have previously identified macroscopically intact ileum as a site of increased intestinal permeability not only in CD but also in UC (15, 17). Although, these studies, by the nature of their methodology, do not provide a mechanistic explanation of increased permeability in macroscopically unaffected ileal mucosa, they strengthen the concept that the ileum is critically involved in disease etiology and disease behavior in UC patients.

Recently, Hiyama analyzed whether the phenotypic appearance of Peyer’s Patches in the terminal ileum, evaluated under narrow-band imaging and magnification endoscopy, is associated with clinical disease behavior. As such, this multicenter study on 105 UC patients in clinical remission was able to demonstrate that the presence of a “Villi Index Low” type was a significant factor for predicting sustained clinical remission (32).

In summary, in this additional analysis of our ERIca trial on the relevance of barrier function in IBD patients, we were able to show that ileal barrier healing is a novel parameter that is highly predictive of the further course of disease in clinically remittent UC with superior predictive capabilities compared to endoscopic and histologic remission. With this, CLE-based assessment of ileal barrier function during routine ileocolonoscopy might be a helpful tool in clinical practice for stratification of UC patients according to their risk for development of complicated disease behavior.

## Data availability statement

The original contributions presented in the study are included in the article/Supplementary material, further inquiries can be directed to the corresponding author.

## Ethics statement

The studies involving humans were approved by ethics committee and nstitutional Review Board of the Medical Faculty of the Friedrich-Alexander University Erlangen-Nuremberg. The studies were conducted in accordance with the local legislation and institutional requirements. The participants provided their written informed consent to participate in this study.

## Author contributions

TR and MN designed the trial. TR, SZ, MW, and JB analyzed study data and images. WU performed statistical analyzes. AH performed pathologic analyzes. RA, TR, and MN discussed and interpreted findings. All authors contributed to the article and approved the submitted version.

## Footnotes

^1^ www.r-project.org

## Supplementary material

SUPPLEMENTARY Figure S1 Endoscopic disease activity under high definition white light endoscopy. Endoscopic disease activity was assessed along the Mayo Endoscopy Score (MES). According to consensus statement, endoscopic remission was defined as a MES≤1 (upper row) while endoscopically active disease was defined as a MES>1 (lower row).

SUPPLEMENTARY Figure S2 Ileal barrier (dys)function under CLE. Watson °I is defined by an intact epithelial barrier without sites of fluorescein leakage (left image). A functional defect of the ileal barrier (Watson °II) is characterized by single cells that lost epithelial integrity, leading to the formation of an epithelial gap which is accompanied by the efflux of fluorescein into the intestinal lumen (middle image). A structural barrier defect (Watson °III) is defined by shedding of multiple neighboring, leading to the formation of microerosions, and the efflux of fluorescein through the site of epithelial damage into the lumen (right image). White arrows: sites of ileal barrier defect.

SUPPLEMENTARY Figure S3 Histopathologic disease activity. For histopathological scoring in UC, Robarts Histopathology Index (RHI) and Nancy Histopathology Index (NHI) as validated histopathological scores were used. Histologic disease remission was defined as a RHI≤3 without lamina propria or epithelial neutrophils or a NHI≤1. Representative histopathology images under 20-fold and 40-fold magnification are shown.

SUPPLEMENTARY Figure S4 Serum zonulin levels in UC patients with and without ileal barrier dysfunction.

## Abbreviations

CD: Crohn’s disease
CLE: Confocal Laser Endomicroscopy
FU: Follow up
IBD: Inflammatory Bowel Diseases
IO-IBD: International Organization for the Study of Inflammatory Bowel Disease
MES: Mayo Endoscopy Score
MAO: Major adverse outcome
MCS: Mayo Clinical Score
MH: Mucosal healing
NHI: Nancy Histological Index
RHI: Robarts Histopathology Index
STRIDE: Selected Therapeutic Targets in Inflammatory Bowel Disease
UC: Ulcerative Colitis
