# Supplementary figures and images for: Healing of the epithelial barrier in the ileum is superior to endoscopic and histologic remission for predicting major adverse outcomes in ulcerative colitis
Source: Front Med (Lausanne). 2023 Oct 10;10:1221449. doi: 10.3389/fmed.2023.1221449 (PMC10595008; doi:10.3389/fmed.2023.1221449)

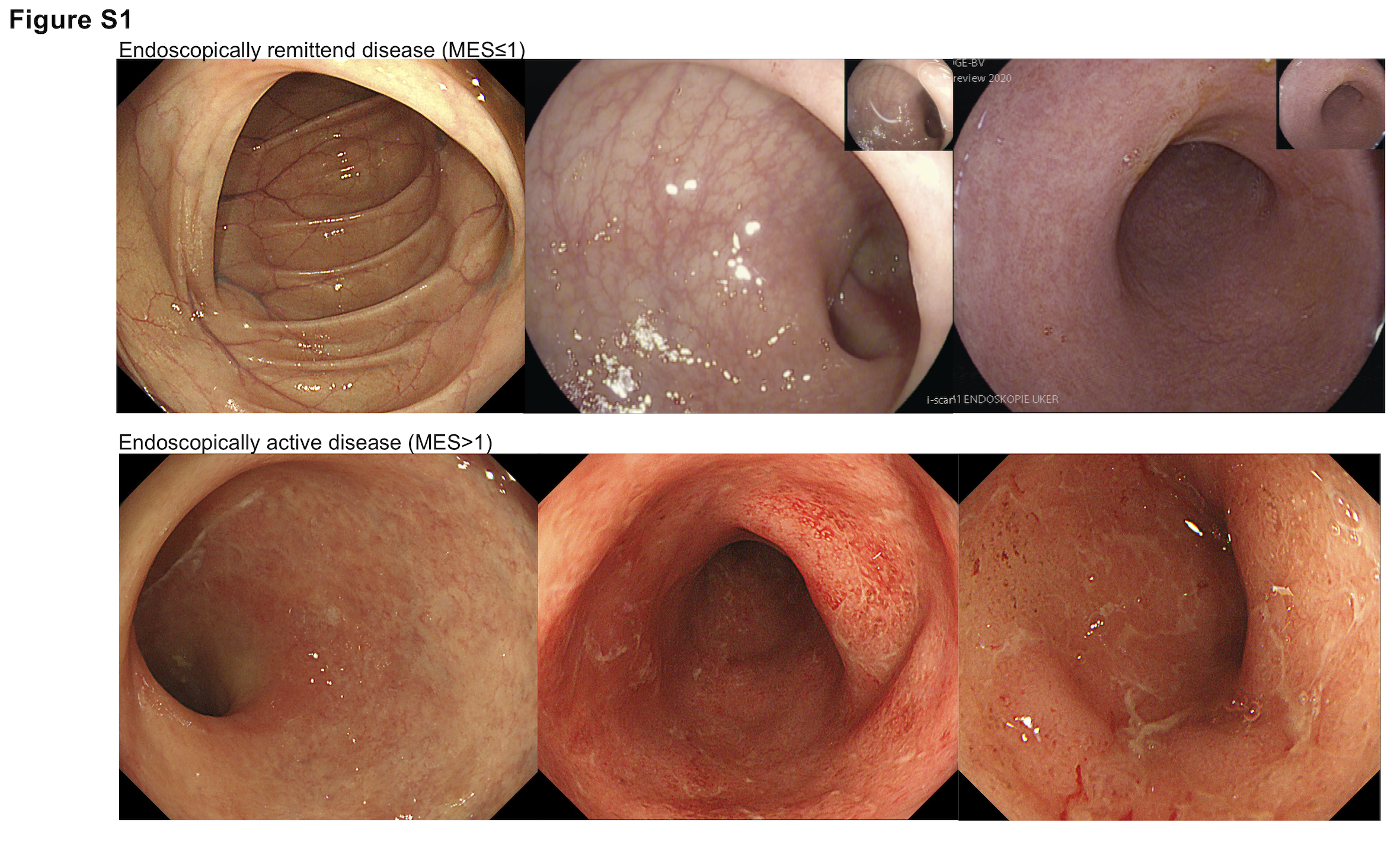

Supplement: SUPPLEMENTARY Figure S1 — Endoscopic disease activity under high definition white light endoscopy. Endoscopic disease activity was assessed along the Mayo Endoscopy Score (MES). According to consensus statement, endoscopic remission was defined as a MES≤1 (upper row) while endoscopically active disease was defined as a MES>1 (lower row). [file Image_1.TIFF]

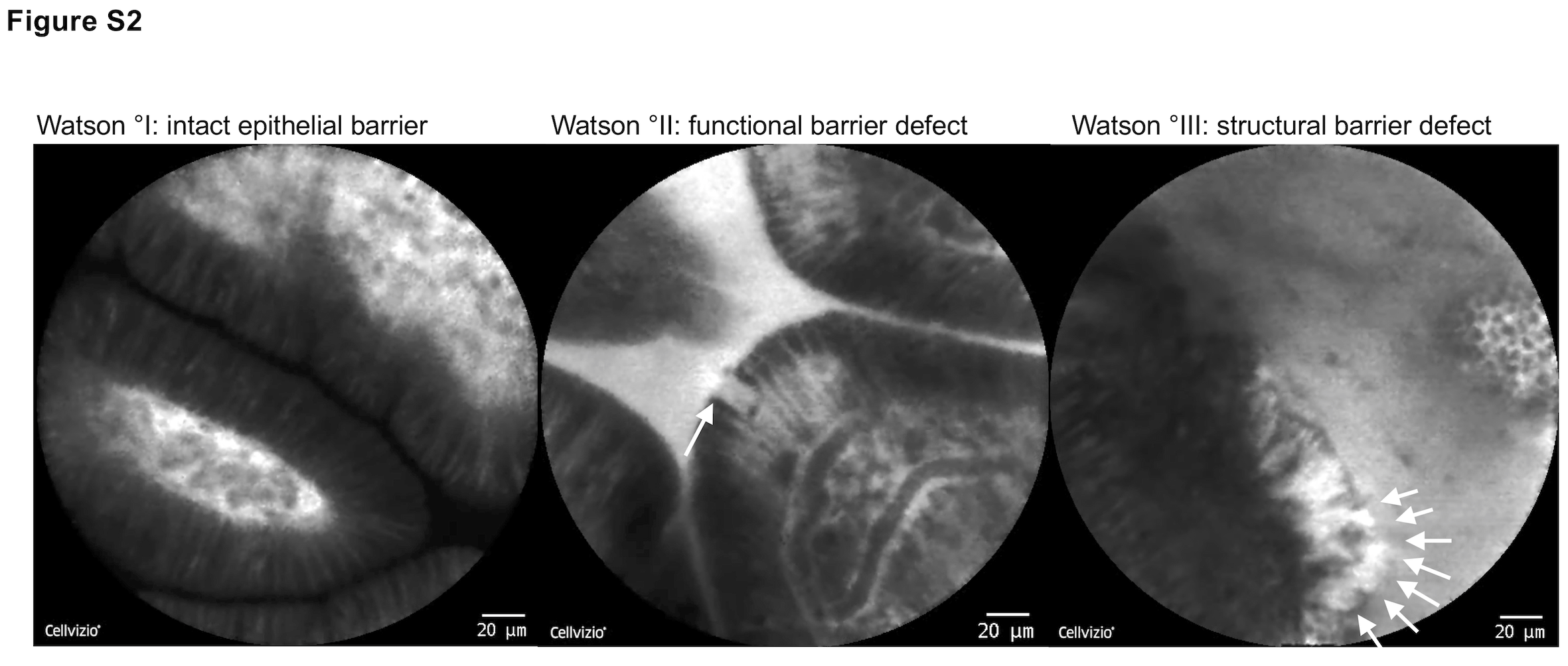

Supplement: SUPPLEMENTARY Figure S2 — Ileal barrier (dys)function under CLE. Watson °I is defined by an intact epithelial barrier without sites of fluorescein leakage (left image). A functional defect of the ileal barrier (Watson °II) is characterized by single cells that lost epithelial integrity, leading to the formation of an epithelial gap which is accompanied by the efflux of fluorescein into the intestinal lumen (middle image). A structural barrier defect (Watson °III) is defined by shedding of multiple neighboring, leading to the formation of microerosions, and the efflux of fluorescein through the site of epithelial damage into the lumen (right image). White arrows: sites of ileal barrier defect. [file Image_2.TIFF]

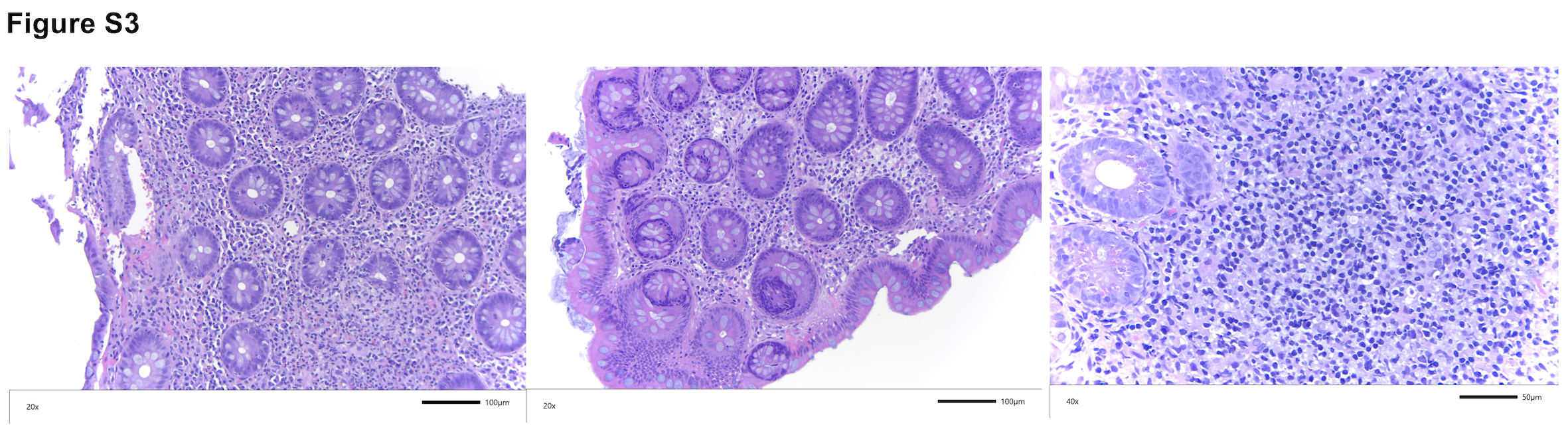

Supplement: SUPPLEMENTARY Figure S3 — Histopathologic disease activity. For histopathological scoring in UC, Robarts Histopathology Index (RHI) and Nancy Histopathology Index (NHI) as validated histopathological scores were used. Histologic disease remission was defined as a RHI≤3 without lamina propria or epithelial neutrophils or a NHI≤1. Representative histopathology images under 20-fold and 40-fold magnification are shown. [file Image_3.TIFF]

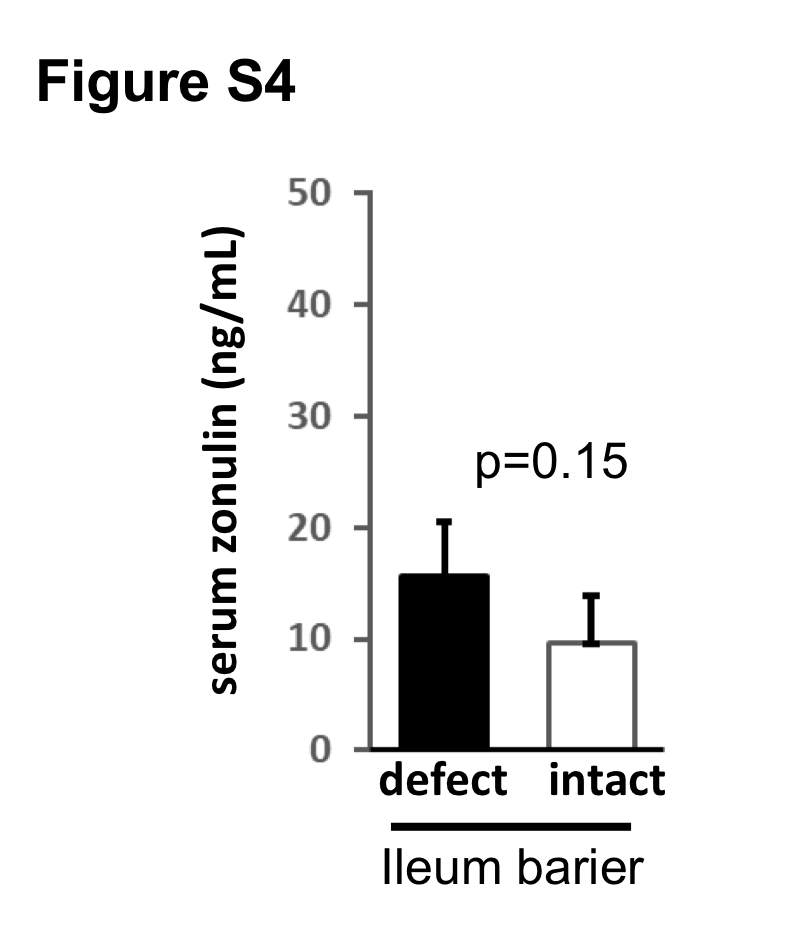

Supplement: SUPPLEMENTARY Figure S4 — Serum zonulin levels in UC patients with and without ileal barrier dysfunction. [file Image_4.TIFF]
