# Supplementary material for: Healing of the epithelial barrier in the ileum is superior to endoscopic and histologic remission for predicting major adverse outcomes in ulcerative colitis
Source: Front Med (Lausanne). 2023 Oct 10;10:1221449. doi: 10.3389/fmed.2023.1221449 (PMC10595008; doi:10.3389/fmed.2023.1221449)
Supplement: Supplementary file 5 [file Table_1.docx]

**Table S1**: Clinical, endoscopic and histologic characteristics of UC patients with and without ileal barrier healing

|  | **UC patients with ileal barrier healing (n=22)** | **UC patients without ileal barrier healing (n=51)** |
| --- | --- | --- |
| **Clinical characteristics** |  |  |
| **Age** (y)  mean, range | 41 (18-67) | 39 (20-77) |
| **Sex** (m/f) | 10/11 | 21/30 |
| **BMI**,  mean, range | 26.6 (20 – 39.2) | 25.2 (17.2 – 38.1) |
| **Disease duration** (y)  mean ± SD | 11.6 ± 9.4 | 8.2 ± 7.1 |
| **Extent of disease**, n (%)  Proctitis  Leftsided colitis  Pancolitis | 0 (0)  13 (59.1)  9 (40.9) | 5 (9.8)  22 (43.1)  24 (47.1) |
| **Medication**, n (%)  **5-ASA derivates**  Mesalazin  **Corticosteroids**  Budesonide  Prednisolone  **Immunomodulator**  6-Mercaptopurin  Azathioprin  **Biological Therapy**  Anti-TNF  Vedolizumab  Tofacitinib  Ustekinumab  **Combination Therapy**  **No medication** | 5 (22.7)  1 (4.5)  0 (0)  0 (0)  1 (4.5)  7 (31.8)  5 (22.7)  0 (0)  0 (0)  1 (4.5)  1 (4.5) | 8 (13.3)  2 (3.3)  3 (5)  0 (0)  4 (6.6)  20 (33.3)  7 (11.6)  3 (5)  2 (3.3)  6 (10  5 (8.3) |
| **Laboratory parameters,** mean ± SD  Leukocyte count (10^9^/L)  C-reactive Protein (mg/L)  Albumin (g/dL)  Hematocrit (%) | 7.7 ± 3.26  3.9 ± 6.6  40.8 ± 4.26  41.9 ± 3.7 | 8.2 ± 3.2  4.8 ± 6.6  39.3 ± 6  41.3 ± 4.2 |
| **Endoscopic and histopathologic data** |  |  |
| **Mayo Endoscopic Score**, n (%)  ≤ 1  > 1  **Barrier Function**, n (%)  **Ileum**  Barrier Healing present  Barrier Healing absent | 19 (86.4)  3 (13.6)  22 (100)  0 (0) | 25 (49)  26 (51)  0 (0)  51 (100) |
| **Histopathology Scoring**, n (%)  RHI ≤ 3  RHI > 3  Nancy ≤ 1  Nancy > 1 | 18 (81.8)  4 (18.2)  18 (81.8)  4 (18.2) | 22 (43.1)  29 (56.9)  21 (41.2)  30 (58.8) |

***RHI****, Robarts Histology Index;* ***Nancy****, Nancy Histological Index*
